# Safety and Efficacy of Colonic Stenting as a Bridge to Surgery: A Retrospective Study

**DOI:** 10.7759/cureus.100603

**Published:** 2026-01-02

**Authors:** Mariam Asad, Muhammad Fahd Shah, Irfan Ul Islam Nasir, Amer Rehman Farooqi, Muhammad Waqas

**Affiliations:** 1 Surgical Oncology, Shaukat Khanum Memorial Cancer Hospital and Research Centre, Peshawar, Peshawar, PAK; 2 Gastroenterology, Shaukat Khanum Memorial Cancer Hospital and Research Centre, Peshawar, Peshawar, PAK

**Keywords:** bridge to surgery, colorectal cancer, large bowel obstruction, self-expanding metallic stents, stent related complications

## Abstract

Objective

The objective of this study was to establish the safety and efficacy of stenting as a bridge to surgery (BTS) by comparing outcomes in patients who underwent stenting followed by surgery with those who proceeded directly to surgery.

Methodology

Data were collected retrospectively for all patients who presented with left-sided malignant colonic obstruction between April 1, 2021, and December 31, 2024, at the Surgical Oncology Department, Shaukat Khanum Memorial Cancer Hospital and Research Centre, Peshawar, Pakistan. In this observational, non-randomized study, all patients of either gender aged 18 years or older with biopsy-proven left-sided colon cancer were included. Patients with metastatic disease were excluded.

Results

A total of 29 patients were included in this study, out of which 22 (75.9%) underwent stenting as a BTS, while seven (24.1%) went straight to surgery without stenting. The most frequent procedure in the stenting group was laparoscopic anterior resection with anastomosis (n = 14); while, in the non-stenting group, Hartmann’s procedure (n = 7) was predominant. Overall, seven patients (24.1%) developed perforation; however, the difference in perforation rates between the two groups was not statistically significant (p = 0.753). Stoma formation was significantly lower in the stenting group (p = 0.018). Key perioperative parameters did not show any statistically significant changes between the stented and non-stented groups.

Conclusions

Colonic stenting is a safe and viable option in patients with left-sided malignant colonic obstruction to decompress the bowel, which results in significantly higher rates of primary anastomosis and avoidance of stoma.

## Introduction

Colon cancer is the third most prevalent cancer internationally, and emergency presentation with obstruction has been reported in 15-30% of cases [1]. Traditional treatment for large bowel obstruction has been emergency surgery, often with a colonic resection and the formation of a stoma [2]. Although effective, this is associated with high mortality ranging from 23% to 45% [3,4] due to old age, pre-existing comorbidities, anesthetic risks, and emergency surgery risks. The stoma itself may be a major source of morbidity, with up to 30% of patients never undergoing reversal [5].

Colon stenting is a minimally invasive procedure that involves the insertion of a self-expanding metallic stent (SEMS) through the tumor to relieve obstruction. SEMS insertion serves as a bridge to allow the patient to recover from acute symptoms of the obstruction and prepare for elective surgery for curative intent [6]. Tejero et al. first reported SEMS as a bridge to surgery (BTS) for malignant colorectal obstruction in 1994 [7]. Since then, several studies have demonstrated stent placement as a safe alternative to conventional emergent surgical management of malignant obstruction of the colon [8,9]. While stenting has benefits, it has potential complications, including stent migration, perforation, and re-obstruction. The overall complication rate associated with SEMS use as a BTS has been reported to be 7-23% [10,11].

We conducted this study to establish the safety and efficacy of colonic stenting by comparing patients who underwent BTS with those who went straight to surgery, focusing on short-term outcomes, including complications of stenting, operative methods, stoma formation, and length of hospital stay.

## Materials and methods

This was a retrospective, observational, non-randomized study carried out at the Surgical Oncology Department, Shaukat Khanum Memorial Cancer Hospital and Research Centre, Peshawar, Pakistan. Data were collected from the hospital information system for all patients undergoing colonic stenting for left-sided malignant bowel obstruction from April 1, 2021, to December 31, 2024. Patients proceeded directly to surgery when stenting was unsuccessful or not readily available. The sample comprised 29 patients who presented with malignant colonic obstruction. Statistical analysis was performed using IBM SPSS Statistics for Windows, Version 25 (Released 2017; IBM Corp., Armonk, New York, United States).

All patients with biopsy-proven left-sided colon cancer presented with obstruction, and those above 18 years of age were included. Patients with metastatic disease were excluded. All stents were placed by an experienced consultant gastroenterologist. The study was approved by the Institutional Review Board (IRB) of Shaukat Khanum Memorial Cancer Hospital and Research Center (SKMCH&RC) (approval no. EX-05-04-25-02).

## Results

**Figure 1:**
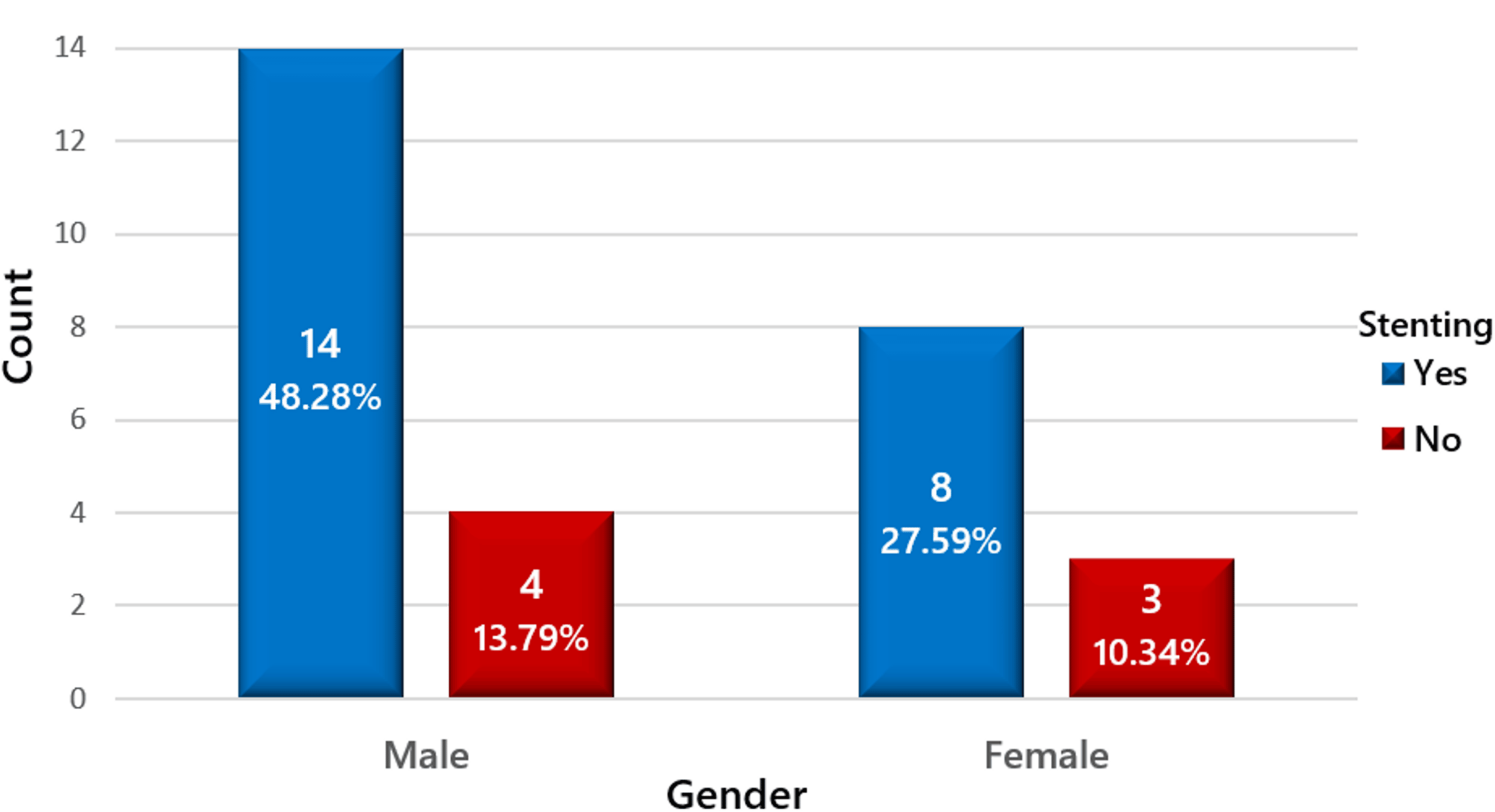
Gender-wise distribution of the study population

A total of 29 patients were included in this retrospective study, comprising 18 males (62.1%) and 11 females (37.9%). The mean age was 44.9 ± 12.06 years, with males averaging 43.1 ± 12.01 years and females 47.8 ± 12.11 years. The distribution of patients on the basis of gender is reflected in Figure 1.

Tumor location and staging are shown in Tables 1-2.

**Table 1:** Tumor location

Tumor location	Stenting as a bridge to surgery	Straight to surgery
Descending colon	1 (4.5%)	0
Sigmoid colon	18 (81.81)	7 (100%)
Rectosigmoid junction	3 (13.6%)	0

**Table 2:** Histological staging

Histology	Stenting as a bridge to surgery	Straight to surgery
II	8 (36.3%)	2 (28.5%)
III	8 (36.3%)	4 (57.1%)
IV	6 (27.2%)	1 (14.2%)

Out of 29 patients, 22 (75.9%) underwent colonic stenting as a BTS, while seven (24.1%) went straight to surgery without stenting. The most common tumor site was the sigmoid colon (86.2%), followed by rectosigmoid and descending colon. The most frequent procedure in the stenting group was laparoscopic anterior resection with anastomosis (n = 14); while, in the non-stenting group, Hartmann’s procedure (n = 7) was predominant.

Overall, seven patients (24.1%) developed perforation. However, the difference in perforation rates between the two groups was not statistically significant (p = 0.753).

This study demonstrated that stenting achieved a technical success rate of 100% (n = 22, radiologically verified stent placement without immediate complications) and an overall symptom relief rate of 63.63% (n = 14, alleviation of obstructive symptoms post-procedure). The findings show that most patients in the stenting cohort achieved successful decompression, facilitating planned elective surgical procedures. Stoma formation was significantly lower in the stenting group (n = 11 (50.0%) in the stenting group vs. n = 7 (100%) in the straight-to-surgery group; p = 0.018). It is worth mentioning that of the 11 patients with stoma formation in the stenting group, three had temporary stomas. There was no significant difference in length of stay between the stenting and non-stenting groups (p = 0.815). In addition, n = 10 (45.5%) of patients who underwent stenting as a BTS experienced stent migration. Detailed outcome analysis is presented in Table 3.

**Table 3:** Comparison of study outcomes (qualitative)

Outcomes		Study groups		P-value
		Stenting as a bridge to surgery	Straight to surgery	
Complications	Perforation	5 (22.7%)	2 (28.6%)	0.072
	Stent migration	10 (45.5%)	0	
	None	7 (31.8%)	5 (71.4%)	
Stoma formation	Yes	11 (50.0%)	7 (100%)	0.018
	No	11 (50.0%)	0	

Key perioperative parameters did not show any statistically significant changes between the stented and non-stented groups, according to the study. The average length of stay was similar for the straight to surgery and stenting group (5.59 ± 2.20 days vs. 4.86 ± 0.69 days; p = 0.397). Likewise, there was no significant difference in the length of the surgery (242.82 ± 66.33 vs. 213.57 ± 32.21 minutes; p = 0.275) or the amount of blood lost during the procedure (38.64 ± 24.70 vs. 30.71 ± 14.27 mL; p = 0.430). Detailed analysis is illuminated in Table 4.

**Table 4:** Comparison of study outcomes (quantitative)

Outcome	Procedure	Mean	Standard deviation (±)	P-value (t-test)
Length of stay (days)	Stenting as a bridge to surgery	5.59	2.20	0.397
	Straight to surgery	4.86	0.69	
Duration of surgery (minutes)	Stenting as a bridge to surgery	242.82	66.33	0.275
	Straight to surgery	213.57	32.21	
Blood loss (mL)	Stenting as a bridge to surgery	38.64	24.70	0.430
	Straight to surgery	30.71	14.27	

Stratified analysis showed no significant differences in immediate postoperative complications or stoma formation between males and females. Higher perforation rate and stoma formation were observed in younger patients compared to older patients. Sigmoid tumors had a significantly higher stoma rate when stenting was performed (p = 0.032).

## Discussion

This study determines the short-term outcomes of patients who underwent stenting as a BTS and those who went straight to surgery, concentrating on three clinical outcomes: complications related to stenting (perforation, stent migration), the necessity for stoma formation, and the average length of hospital stay. The results elucidate the significance of stenting in surgical decision-making and postoperative care. The incidence of perforation was somewhat higher in patients who did not have stenting (28.6%) in comparison to the stenting as a BTS group, and this was due to the advanced disease; however, this disparity was not statistically significant.

The findings indicate that stenting does not markedly affect the risk of perforation within the examined cohort, as no significant difference has been observed. A study conducted by Kim et al. also endorsed these findings [12]. Nonetheless, considering that 25% of patients in the study of Ho et al. [13] encountered technical stent failure, procedural success is a crucial factor in ensuring safe results. In a multicenter trial by Pirlet et al. [14], the technical failure rate for stenting was 53.3%, highlighting the diversity in operator expertise and equipment accessibility. The absence of a notable correlation may stem from the limited sample size or the generally low occurrence of problems, which constrains the ability to identify a genuine disparity. On the other hand, the observed trend of reduced perforation rates in the stented group may be clinically significant, reinforcing the idea that stenting can function as a BTS strategy, alleviating surgical urgency and facilitating improved patient optimization [15]. A statistically significant correlation was identified between stenting and the necessity for stoma creation. Every patient in the non-stented group (100%) necessitated a stoma, but only 50% of patients in the stented group did (p = 0.018), because emergency operations for malignant obstruction typically require diverting stomas to avoid anastomotic leaks in unprepared bowel.

This study corresponds with current literature indicating that preoperative stenting in obstructive colorectal cancer may diminish the necessity for a diverting or permanent stoma by transforming an emergency treatment into a planned elective one [16]. The 50% reduction in stoma formation with stenting mirrors the results of Arezzo et al.’s meta-analysis of eight randomized controlled trials (RCTs), where stenting reduced temporary stoma rates by 33% (relative risk (RR) 0.67, p < 0.001) and permanent stoma rates by 34% (RR 0.66, p = 0.003) compared to emergency surgery [17]. This benefit is particularly pronounced in sigmoid tumors, reinforcing anatomical suitability as a critical factor. The lack of substantial differences indicates that stenting does not increase procedural risks, which is a crucial consideration for its use as a BTS, even though it did not shorten the length of stay, operating time, or blood loss when compared to emergency surgery. Although the stented group's statistically longer surgical time (242.82 vs. 213.57 minutes) did not result in worse clinical outcomes, it might be a reflection of the extra time taken in laparoscopic surgery, as well as the allowance of training opportunity in elective surgery when compared to emergency surgery. The safety of stenting before resection is supported by the comparable blood loss between groups (p = 0.430). These results are consistent with earlier research [18] and support the idea that the main advantages of stenting are elective surgery and a decrease in stoma rates.

Our findings are more conclusive than those presented in prior RCTs. Ho et al. observed a reduced, although statistically insignificant, stoma rate in the stenting cohort relative to emergency surgery. Pirlet et al. similarly found no statistically significant difference in stoma formation rates between the stented group and the surgical group. The Dutch study [19], interestingly, revealed a markedly reduced stoma rate immediately following the initial intervention in the stenting group (p = 0.016); however, long-term stoma rates showed no significant difference. The varied findings in the literature indicate that although stenting may diminish the immediate necessity for a stoma, this advantage is not uniformly observed across different contexts, possibly attributable to variations in clinical protocols, scheduling of elective surgeries, and problems associated with stents. Our findings underscore the efficacy of stenting in preventing stomas when effective decompression is attained. The notable decrease in stoma formation among stented patients highlights a key therapeutic benefit of stenting. It enables primary anastomosis without diversion in a greater percentage of patients. This affects both surgical results and patient quality of life, as well as long-term healthcare expenses [20].

Despite lacking statistical significance (p = 0.815), a trend was seen indicating a shorter or comparable length of hospital stay for patients who received stenting. The majority of patients, regardless of stenting status, experienced a hospital stay lasting four to six days. A marginally greater percentage of patients in the stented cohort were discharged within four days (27.3%) than those in the non-stented cohort (28.6%). Nonetheless, 57.1% of non-stented patients remained hospitalized for five days, as contrasted to 36.4% of the stented cohort, suggesting a slight difference in duration of stay that lacked statistical significance. The extensive distribution and limited quantities within each category likely led to the absence of significance. Nonetheless, stenting may facilitate a more stable postoperative trajectory, potentially allowing for quicker recovery and discharge in chosen individuals [21].

## Conclusions

The findings of this study endorse the application of preoperative stenting in suitable colorectal scenarios, especially for diminishing the probability of stoma creation, a vital element in enhancing patient satisfaction and mitigating long-term morbidity. Although no notable difference was detected in complication rates or duration of hospitalization, the clinical trends suggest that stenting may improve perioperative outcomes by facilitating planned, elective surgeries and alleviating the physiological strains and logistics linked to emergency procedures. This study is constrained by its short sample size, which diminishes the statistical power of the analyses and may result in type II errors. Moreover, it was performed at a single center, which may restrict generalizability. The study failed to stratify patients according to tumor stage or comorbidities, which may serve as confounding variables. Subsequent research with expanded sample sizes and multicenter data is essential to corroborate these findings.
